# Integrating oncolytic adenoviruses into combination cancer therapy: Mechanisms, advances and clinical outlook

**DOI:** 10.1002/ctm2.70702

**Published:** 2026-05-23

**Authors:** Vlad Iova, Gilda Mihaela Iova, Mihail Silviu Tudosie, Ioana Scrobota, Catalin Gabriel Smarandache

**Affiliations:** ^1^ Faculty of Medicine ‘Carol Davila’ University of Medicine and Pharmacy Bucharest Bucharest Romania; ^2^ Department of Dental Medicine Faculty of Medicine and Pharmacy University of Oradea Oradea Romania; ^3^ ICU II Toxicology Clinical Emergency Hospital Bucharest Romania; ^4^ 4th Department of Surgery, Bucharest Emergency University Hospital Bucharest Romania; ^5^ Cantacuzino National Military Medical Institute for Research and Development Bucharest Romania

**Keywords:** adenoviruses, combination cancer therapy, oncolytic viruses, virotherapy

## Abstract

**Background:**

Cancer represents a major global public health issue, and recent research has focused on improving the efficacy of existing therapeutic approaches. In this context, oncolytic viruses have emerged as promising treatment options for advanced and treatment‐resistant malignancies. Among them, adenoviruses are the most extensively studied due to their favourable safety profile, biological versatility and ease of genetic modification to enhance therapeutic performance.

**Main body:**

Combination‐based strategies integrating virotherapy with chemotherapy, radiotherapy or immunotherapy have been widely investigated to further improve anti‐tumour efficacy. These approaches act through complementary and synergistic mechanisms, where chemotherapy can promote viral replication and increase tumour cell susceptibility through deoxyribonucleic acid (DNA) damage and transient immunomodulation, while radiotherapy enhances immunogenic tumour cell death by inducing sustained DNA damage. In parallel, immunotherapy—particularly immune checkpoint inhibition—can strengthen anti‐tumour immunity by reversing T‐cell exhaustion and increasing CD8^+^ T‐cell infiltration and effector function. Collectively, these interactions amplify both direct viral oncolysis and immune‐mediated tumour clearance. Additionally, clinical trial data suggest that intravenous administration of oncolytic adenoviruses may provide greater systemic antit‐umour activity compared with intratumoural delivery, albeit with increased toxicity relative to localised injection.

**Conclusion:**

Oncolytic adenoviruses have emerged as promising treatment options for advanced and treatment‐resistant malignancies, particularly when integrated with chemotherapy, radiotherapy or immunotherapy. However, most studies included are early‐phase trials conducted in small patient cohorts, underscoring the need for further research to better define safety profiles, long‐term adverse effects, and the underlying mechanisms driving therapeutic response.

## Key points

- Oncolytic viruses have been proposed as alternatives to standard anti‐cancer treatments for improved outcomes.
- Oncolytic adenoviruses work through synergy with standard therapies (chemo‐, radio‐, immunotherapies), overcoming tumour‐associated resistance, refining delivery and systemic toxicity and optimising cell‐based therapies.
- Only one oncolytic adenovirus has been approved for clinical use (H101, Oncorine), and the majority of clinical trials are of early phase.

## Graphical abstract

Oncolytic adenoviruses synergise with chemotherapy, radiotherapy and immunotherapy to enhance anti‐tumour efficacy. Chemotherapy and radiotherapy increase tumour susceptibility and antigen release through DNA damage, while immunotherapy restores T‐cell activity. Together, these effects amplify viral oncolysis and immune‐mediated tumour clearance, resulting in improved local and systemic tumour control.

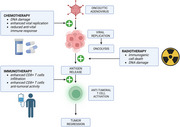

## 1 INTRODUCTION

Cancer represents a constant threat to public health because it constitutes the second leading cause of mortality worldwide.^1^ Even though cancer research has been constantly advancing, the need to improve the understanding of tumour biology remains, as novel treatments are studied.^1^ As a result, oncolytic viruses (OVs) have been proposed as new and promising alternatives for patients with resistant cancers.^2^ OVs appear due to natural evolution or may be engineered by altering existing viruses.^3^ The most studied classes of OVs are herpes simplex virus, adenovirus (Ad), influenza virus, vaccinia virus and coxsackievirus.^4^ Only four OVs have been approved for cancer treatment, namely Rigvir (ECHO‐7) in Latvia for melanoma, Oncorine (H101) in China for head and neck cancer, T‐VEC (Imlygic) in the United States and Europe for melanoma, and DELYTACT (Teserpaturev/G47∆) in Japan for malignant glioma.^5^

Ads, part of the Adenoviridae family, are nonenveloped viruses with a deoxyribonucleic acid (DNA) genome and constitute one of the most frequently employed classes of OVs.^6^, ^7^ Currently, oncorine (H101) is the sole Ad approved for anti‐cancer use.^5^

The lower‐than‐expected effectiveness of several OVs results from their limited ability to infect some tumour types, their lack of selectivity, their poor dissemination within tumours and their attack by the host immune response.^8^ In order to address these problems, different combination‐based approaches have been tested, including those with chemotherapy, targeted therapy, radiotherapy, immunotherapy or even other oncolytic viruses.^8^, ^9^, ^10^, ^11^

The purpose of this review is to provide an integrated analysis of clinical trials investigating combination therapies based on adenoviral vectors, with a focus not only on their safety and efficacy but also on identifying cross‐trial patterns that inform therapeutic optimisation. Furthermore, we comparatively evaluated different combination strategies and routes of administration, highlighting how specific viral design features and treatment partners influence clinical outcomes, toxicity profiles and systemic versus local responses. This synthesis aims to generate clinically relevant insights to guide the rational design of future trials. We chose to treat oncolytic Ads because they show an enhanced safety profile, unique biological properties, and flexibility for genetic modification to improve their effectiveness.^12^

A structured search of the ClinicalTrials.gov database was performed to identify relevant clinical trials investigating oncolytic viruses in combination with cancer therapy. The search was conducted using the term ‘oncolytic virus’ in the ‘Intervention/Treatment’ field, without additional filters or restrictions. This initial query yielded 236 records. Trials were subsequently screened manually to include only those involving oncolytic adenoviruses administered in combination with other therapeutic modalities (e.g., chemotherapy, radiotherapy, immunotherapy or targeted therapy). Based on these criteria, 30 clinical trials were selected for inclusion. In addition, one relevant study not captured in the initial search was identified through complementary database screening and included.

## 2 ONCOLYTIC ADS IN COMBINATION THERAPY: MECHANISMS, PRECLINICAL AND CLINICAL EVIDENCE

### 2.1 Viral engineering strategies

**FIGURE 1:**
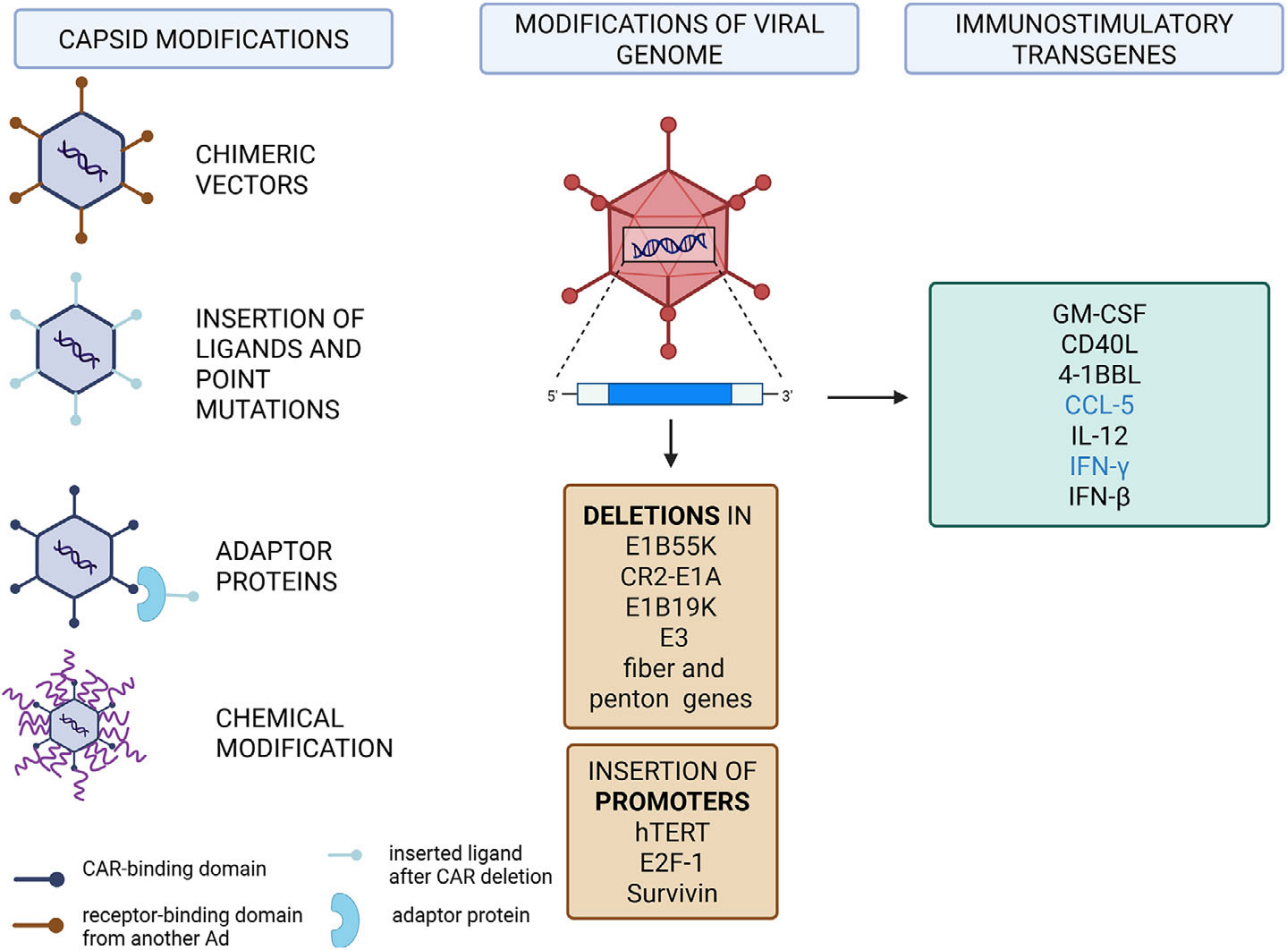
Ways to improve the efficiency of oncolytic adenoviruses. These changes include capsid modifications (chimeric vectors, insertion of ligands or point mutations, the use of adaptor proteins and chemical modifications, leading to enhanced tumour tropism), modifications of the viral genome (deletion in viral genes and insertion of tumour‐specific promoters) and transportation of immunostimulatory transgenes (leading to an increased anti‐tumoural immune response at the action site). E1B55K deletion enables selective replication in p53‐deficient tumour cells; CR2‐E1A deletion allows selective replication in cancer cells with defective Rb pathway; E1B19K deletion leads to apoptosis of infected cells; deletions in E3 region remove specific immune‐modulatory genes; deletions in fibre/penton genes alter tropism and improve safety; insertion of hTERT, E2F‐1 and surviving promoters further enable tumour tropism by selective replication in cancer cells with altered molecular pathways. Insertion of CCL‐5 and IFN‐γ transgenes is still in the preclinical phase and is marked with blue.^13^ Created in BioRender, Iova V. (2026); https://BioRender.com/b0onxiy.

Over the years, oncolytic Ads have been modified for better tumour tropism, selectivity and anti‐tumour efficacy, changes that include capsid modifications, small deletions in the pivotal viral genes, insertion of tumour‐specific promoters and addition of immunostimulatory transgenes.^6^ These modifications are summarised in Figure 1.

The genome of the human Ad consists of 38 kilobases (kb). The virus is encapsulated by an icosahedral nucleocapsid made of nine proteins, of which hexon, penton base and fibre are referred to as major capsid proteins.^7^, ^14^, ^15^, ^16^ This capsid is pivotal for viral transduction through attachment of the fibre protein to its primary receptor (coxsackievirus and adenovirus receptor [CAR] for most species) and interaction of cellular integrins with the Arg‐Gly‐Asp (RGD) sequence from the penton base.^17^ However, the natural target of the virus is rarely represented by the correct target needed for therapeutic outcome (cancer cells, in this case).^7^ Hagedorn et al. reviewed capsid modifications required for tumour tropism: the use of chimeric vectors, directed evolution, insertion of peptides and point mutations (such as ablation of CAR‐binding domains), single‐component adapters and chemical modification.^7^

The adenoviral genome consists of four early genes (E1–E4), allowing the multiplication of Ad, and five late genes (L1–L5), generating capsid proteins.^18^ Like other DNA viruses, Ad has sufficient genome insertion capacity, allowing the transportation of therapeutic genes of about 30–38 kb.^18^ Up to 7.5 kb can be added by deleting viral genes such as the E1 or E3.^18^, ^19^ Small deletions in the pivotal viral genes of common oncolytic Ad vectors are: *E1B‐55K* (which normally binds and inactivates p53; its deletion reduces inhibition of p53‐dependent apoptosis and lead to selective replication in tumour cells with defective p53 while sparing normal cells), *conserved region 2 (CR2) of E1A* (a small 24‐bp deletion allowing viruses to replicate only in cells with defective retinoblastoma [Rb] pathway), *E1B19K* (which is anti‐apoptotic gene, analogous to Bcl2; its deletion leads to apoptosis of infected tumour cells), *small deletions in E3 region* (targeted small deletions to remove specific immune‐modulatory genes) and *short deletions in the fibre and penton protein regions* (altering tropism and improving safety).^18^, ^20^, ^21^, ^22^, ^23^ Another modification is the insertion of tumour‐specific promoters, such as the human telomerase reverse transcriptase (*hTERT*) promoter (hTERT being highly active in immortalised cell lines, like 85% of human cancer cells, but silent in most normal tissues) and the *E2F1* promoter controlling E1A transcription (E2F1 promoter is active in cells with deregulated Rb/E2F signalling, common in many cancers).^24^, ^25^

Moreover, the enhancement of tumour‐specific immune response is a promising approach to increase OVs’ efficiency, and this could be achieved through the addition of immunostimulatory transgenes coding for molecules such as granulocyte‐macrophage colony‐stimulating factor (GM‐CSF), CD40L, tumour necrosis factor superfamily member 9 (4‐1BBL), C–C motif chemokine ligand 5 (CCL‐5), interleukin (IL)‐12 and interferon (IFN)‐γ.^26^, ^27^, ^28^, ^29^

### 2.2 Preclinical evidence

Several preclinical studies have proved the potential efficiency of various Ads, like OAd35, AdV‐SIRPα/Siglec‐10, ADVNE, ADVPPE, CAdTetra, OAd‐B7H3‐BiTE, TILT‐123, Ad5‐hTERT, BSV, ADV‐ApoA1, AdCab, RCAd‐LTH‐shPD‐L1 and CaP‐OMV@P_2_O‐Ads, on different cancer models, such as melanoma, hepatocellular carcinoma, colorectal carcinoma, ovarian cancer, pancreatic cancer, bladder cancer and glioblastoma. They generally act by increasing the number of anti‐tumoural cells (like NK cells, CD8^+^ T cells, M1 macrophages or neutrophils) and cytokines (IFN‐I, IFN‐γ, HMGB1, IL‐6, IL‐12) or other cytotoxic proteins (granzyme B, perforin). They also induce the expression of MHC‐I and ‐II proteins on antigen‐presenting cells and enhance the process of tumour cell autophagy, increasing the replicative rate of Ads. Additionally, they lower the number of cells related to a low immune response within the tumour microenvironment (TME), such as M2 macrophages and exhausted CD8^+^ T cells. Furthermore, the products of these Ads can mark various tumour‐associated antigens (like CD24, CD44v6, CD47, B7H3 and MUC1) for recognition by the immune system. Consequently, these Ads may redirect tumour‐infiltrating T cells against cancer cells. Additionally, these products can target molecules essential for tumour survival (like mammalian target of rapamycin‐mTOR or signal transducer and activator of transcription 3‐STAT3), proteins that lead to immunosuppression (like programmed death‐ligand 1‐PD‐L1) or other markers of tumour angiogenesis (such as CD31 and vascular endothelial growth factor [VEGF]). Moreover, some Ads may carry the gene for apoA1 expression, which is a cholesterol reverse transporter that mediates cholesterol efflux from the TME, having anti‐cancer properties.^30^, ^31^, ^32^, ^33^, ^34^, ^35^, ^36^, ^37^, ^38^, ^39^, ^40^, ^41^, ^42^, ^43^, ^44^

Nevertheless, the efficiency of oncolytic Ads is affected by limited ability to infect some tumour types, lack of selectivity, poor dissemination within tumours and attack by the host immune response.^8^ In order to address these problems, different combination‐based approaches have been tested. Combining oncolytic Ads with standard chemotherapeutic drugs and molecularly targeted drugs constitutes an appealing strategy to increase their potency.^8^ Additionally, there is evidence for the potential co‐operation between oncolytic Ads and radiotherapy.^9^ There are also possibilities to combine oncolytic Ads with other immunotherapies, such as immune checkpoint inhibitors (ICPIs), as Ads can reverse tumour resistance towards them.^10^ Additionally, the combination of Ads with other OVs might enhance anti‐tumour efficacy through the induction of tumour‐specific immunity.^11^

**FIGURE 2:**
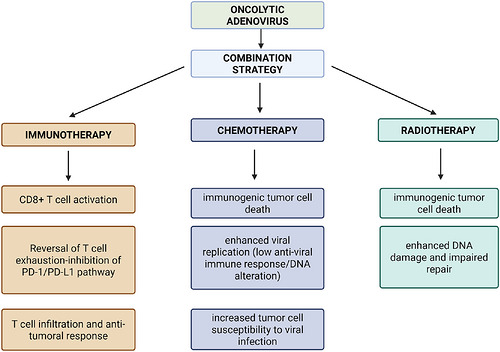
Synergistic anti‐tumoural mechanisms of oncolytic adenoviruses combined with chemotherapy, radiotherapy, and immunotherapy. Combination therapies enhance the efficacy of oncolytic adenoviruses through complementary mechanisms. Chemotherapy promotes viral replication and tumour cell susceptibility via DNA damage and transient immunosuppression. Radiotherapy increases immunogenic tumour cell death by inducing and sustaining DNA damage. strengthens anti‐tumour immunity by reversing T‐cell exhaustion and enhancing CD8^+^ T‐cell infiltration and activity. Together, these approaches amplify viral oncolysis and immune‐mediated tumour clearance. Created in BioRender, Iova V. (2026); https://BioRender.com/0i1nuh6.

The efficiency of oncolytic Ads with various modifications in combination‐based approaches was proved in an array of preclinical studies. The majority of these studies were conducted combining oncolytic Ads with immunotherapy. They can be combined with ICPIs and increase their immunostimulant properties in a wide variety of solid cancers in preclinical models, such as non‐small cell lung cancer (NSCLC), triple‐negative breast cancer (TNBC), colon cancer, glioblastoma and melanoma. These oncolytic Ads act by inhibiting tumour‐associated immunodeficient pathways, like SAM and HD domain‐containing protein 1 (SAMHD1) (oAd‐vpx) or tumour necrosis factor receptor 2 (TNFR2) (AdV‐TNFi, AdV‐amTR2), reducing immunodeficiency‐related cells, like myeloid‐derived suppressor cells (MDSCs) (OAd.shDNMT1, AdV‐TNFi), inducing the expression of tumour‐specific antigens, like epidermal growth factor receptor variant III peptide (PEPvIII) (Ad5‐D24‐PEPvIII) and upregulating the synthesis of proinflammatory cytokines, like GM‐CSF (Ad5‐D24‐PEPvIII) or IL‐18 (OAdDR18). The combination of ICPIs and these Ads acts mainly by triggering CD8^+^ T‐cell‐dependent anti‐tumour immune responses, and has been proven not to have significant toxicities in vivo.^33^, ^35^, ^45^, ^46^, ^47^ Oncolytic Ads were also studied in preclinical models of glioblastoma along with chimeric antigen receptor T‐cell‐based approaches (CAR‐T cells). oAd‐CXCL11 leads to the expression of C–X–C motif chemokine ligand 11 (CXCL11) in the TME, leading to the infiltration of associated CAR‐T cells that target the tumour‐specific B7H3 antigen. This combination results in a potent anti‐tumour effect with a durable response.^48^ YSCH‐01 is an oncolytic Ad that was proven to be effective against glioblastoma in vivo in combination with anti‐IL‐8 therapies (such as reparixin or glucocorticoids). IL‐8 is secreted in the TME following viral replication in the tumour cells, leading to the formation of a barrier of senescent and fibrotic cells, halting viral propagation. Thus, the addition of anti‐IL‐8 molecules inhibits the formation of this barrier following viral replication, allowing YSCH‐01 to successfully attack neighbouring tumour cells.^49^ Additionally, OAd‐based treatments can be used together with the administration of thymosin α1. Oncolytic Ads were proven to increase the number of tumour‐associated macrophages with the M2 phenotype and of regulatory T cells, both having immunosuppressive properties. It was proven both in vitro and in vivo that the addition of thymosin α1 following oncolytic Ad administration induces the generation of an anti‐tumoural phenotype of macrophages, increasing anti‐tumoural immunity. Thus, the anti‐tumoural effects of oncolytic Ads through the infiltration of CD8^+^ T cells are enhanced.^50^ Moreover, it was also preclinically shown that oncolytic Ads can also be used along with chemotherapy. Chemotherapeutic agents can suppress antiviral immune responses (e.g., cyclophosphamide), thereby prolonging viral persistence and increasing intratumoural viral replication. Other drugs sensitise tumour cells to viral oncolysis by inducing DNA damage, disrupting cell cycle progression, or inhibiting DNA repair pathways, which facilitates more efficient viral replication and cell killing. In parallel, some agents improve viral spread by increasing tumour cell permissiveness to infection. Conversely, oncolytic Ads can enhance chemotherapy efficacy by promoting tumour cell death pathways such as apoptosis or autophagy and by overcoming resistance mechanisms. Overall, the synergy arises from chemotherapy enhancing viral delivery, replication, and persistence, while the virus amplifies cytotoxic and immunogenic effects within the tumour.^51^ For instance, in human and murine colorectal cancer models, AdV‐ATA47 replication within cancer cells leads to the production of ATA47, a bispecific antibody fusion protein targeting both CD47 (an antigen that is overexpressed in colorectal cancer and takes part in immune evasion mechanism) and TNFR2 (which activates a tumour‐associated immunosuppressive pathway), leading to an enhanced tumour control and anti‐tumoural immune response, with low systemic toxicity. This fusion protein acts by lowering the number of regulatory T cells and MDSCs and increasing the number of CD8^+^ T cells. Consequently, AdV‐ATA47 has been proposed as a promising therapeutical approach in colorectal cancer, particularly in combination with conventional colorectal cancer therapies.^52^ An interesting combination regimen was proposed using Adv‐MCK and photodynamic therapy in pancreatic cancer. Adv‐MCK leads to the expression of the KillerRed gene, producing a photosensitiser protein. Consequently, this regimen improves the hypoxia within TME and activates anti‐tumour immunity by recruiting γδ‐T cells.^53^ Furthermore, it has been proven that on mouse breast cancer models, in situ placement of an Ad‐loaded hydrogel (adv@Nap gel) following tumour resection activates anti‐tumoural immunity and reduces the risk of post‐resection recurrence and metastasis by inducing the IFN‐I pathway.^54^ Oncolytic Ads have also been studied combined with radiotherapy. Radiotherapy and oncolytic Ads exhibit complementary and potentially synergistic anti‐tumour effects, primarily through enhanced radiosensitisation and immune activation. Oncolytic Ads can impair tumour cell DNA repair mechanisms—particularly by inhibiting key components such as the MRE11‐Rad50‐Nbs1 complex and DNA ligase IV—thereby increasing the susceptibility of cancer cells to radiation‐induced DNA damage. This results in augmented cytotoxicity specifically within tumour tissue, while maintaining limited effects on normal cells. In addition, both modalities promote immunogenic tumour cell death and proinflammatory signalling within the TME, which may enhance local and systemic anti‐tumour immune responses.^9^ The mechanisms by which the combinations of oncolytic Ads with immunotherapy, chemotherapy or radiotherapy enhance the anti‐tumoural efficacy of monotherapies are summarised in Figure 2.

### 2.3 The most important oncolytic Ads studied

#### 2.3.1 H101

H101, also known as Oncorine, is an attenuated Ad5 vector approved in China for the treatment of head and neck carcinoma, typically in combination with chemotherapy.^22^ H101 contains genome modifications, giving it selective targeting properties and enhanced immunotherapeutic capabilities, improving its efficiency for virotherapy.^22^ The E1B‐55K region is deleted, leading to selective replication only in p53‐deficient cancer cells.^55^ Additionally, there are four deletions in the E3 region: E3‐11.6 K (whose deletion spares normal tissues from viral spread), E3‐19 K (whose deletion enhances anti‐tumoural immunity), E3‐10.4 K/14.5 K (whose deletion restores apoptosis, leading to cancer cell death) and E3‐14.7 K (whose deletion leads to tumour necrosis factor [TNF]‐mediated cell death of infected cells).^55^, ^56^, ^57^ Many clinical trials and preclinical studies proved the efficiency of H101, either in monotherapy or in combination therapy, in leading to regression of different malignancies: gynecologic, gastric, hepatic, head and neck, oesophagal, lung, glioblastoma, bladder.^58^, ^59^, ^60^, ^61^, ^62^, ^63^ In terms of associated adverse reactions, the most common is a low‐grade, self‐limiting pyrexia.^64^ Other side effects include injection site pain, flu‐like symptoms, nausea or vomiting, fatigue, haematological effects and mild hepatic dysfunction.^60^, ^65^, ^66^, ^67^

#### 2.3.2 LOAd703

LOAd703 (delolimogene mupadenorepvec) is an oncolytic Ad (serotype 5/35) that carries transgenes CD40L and 4‐1BBL, activating antigen‐presenting cells and T lymphocytes.^68^ The property that makes this virus special is the ability to infect B cells via CD46, thus representing a promising approach for B‐cell malignancies.^68^ Replication is restricted to tumour cells by the presence of the 24‐bp deletion in the E1A gene.^69^ Additional modifications include E2F promoters for E1A, and the immune regulatory E36.7K and E3gp19K genes deletion for increased immunogenicity.^70^, ^71^, ^72^ This Ad was proven effective in preclinical studies against various types of tumours, such as multiple myeloma, B‐cell lymphoma or pancreatic cancer.^68^, ^70^, ^73^

#### 2.3.3 CRAd‐S‐pk7

CRAd‐S‐pk7 is a conditionally replicative Ad (CRAd) with a human survivin promoter (S), which drives E1 expression and a polylysine sequence modification of the fibre protein (pk7). This virus can selectively target glioma tissue by binding the overexpressed heparan sulfate proteoglycans through the pk7 modification.^74^, ^75^, ^76^ Survivin is a member of the inhibitor of apoptosis protein family that is typically present during embryogenesis and overexpressed in malignant tissues, including gliomas. Otherwise, it is undetectable in normal tissues.^77^ Consequently, by incorporating survivin as a promoter within the E1A region of the virus, replication is restricted to malignant tissues, such as malignant glioma.^77^, ^78^ Some preclinical studies proved the efficiency of this Ad mainly against glioma models^74^, ^79^, ^80^, ^81^ Neural stem cell (NSC)‐based carriers have the potential to improve the clinical efficacy of virotherapy against glioma by not only protecting therapeutic virus from the host neutralising immune response, but also by increasing the therapeutic payload at tumour sites.^79^ However, Mooney et al. went further to test the delivery of CRAd‐S‐pk7 by allogeneic, clonal NSCs to tumours within preclinical peritoneal ovarian metastases models, as survivin is highly expressed in ovarian cancer too.^82^ The authors suggested that NSC‐delivered CRAd‐S‐pk7 virotherapy held promise for improving clinical outcome, reducing toxicities and improving quality of life for patients with advanced ovarian cancer.^82^

#### 2.3.4 DNX‐2401

DNX‐2401 (Ad5‐Δ24‐RGD, Tasadenoturev) is an oncolytic Ad5 virus containing an integrin binding RGD‐4C motif, allowing CAR‐independent infection of tumour cells.^83^ This modification is important, as integrins are overexpressed in a wide range of tumours, including glioma cells.^84^ In addition to the oncolytic effect, following DNX‐2401 administration, a high level of infiltrated immune cells were noticed in the TME, suggesting an enhanced immune‐mediated antiglioma response.^85^ This virus was proved effective in various glioma preclinical models.^84^, ^86^ The most frequent adverse events of DNX‐2401 monotherapy are fatigue, headache and seizures consistent with pre‐existing symptoms, underlying disease and/or surgery in the treatment of recurrent glioblastoma.^87^

#### 2.3.5 TILT‐123

TILT‐123 (Ad5/3‐E2F‐d24‐hTNFa‐IRES‐hIL2 or igrelimogene litadenorepvec) is a chimeric Ad based on Ad5 with a fibre modification from Ad3, which is used to increase the tumour cell targeting through desmoglein‐2.^88^, ^89^, ^90^ The E2F promoter and the 24‐bp deletion in CR2 of E1A restrict viral replication to cancer cells. The virus encodes human TNFα and IL‐2.^88^ TILT‐123 was designed to enable and enhance T‐cell therapies and ICPIs in solid tumours.^91^ It has shown complete responses in animals treated in combination with tumour‐infiltrating lymphocytes, CAR‐T cells or ICPIs.^91^, ^92^, ^93^, ^94^ TILT‐123 addressed one of the most important limitations of oncolytic virotherapy, namely the requirement for intratumoural administration. Thus, it was proved that TILT‐123 may be used through intravenous delivery because of the capsid modifications. This Ad was well‐tolerated, without any systemic toxicities, and led to an enhanced immune anti‐tumoural response following intravenous administration due to circulating lymphocytes infiltration in the TME.^95^, ^96^ The most frequent associated adverse events were fever, chills and fatigue. The anti‐tumoural response was noticed both at the primary tumour site and at metastases.^96^

#### 2.3.6 ONCOS‐102

ONCOS‐102 is an Ad5 with multiple modifications, including a chimeric 5/3 fibre‐knob region to augment infectivity, a 24‐bp deletion in the E1A region conferring selective replication in tumour cells, and expression of GM‐CSF to increase anti‐tumoural immunity.^97^ It has been shown to be well tolerated in the Phase 1 trial NCT01598129.^48^ ONCOS‐102 leads to increased infiltration of CD8^+^ T cells in the TME and upregulated immune‐related gene expression.^98^, ^99^ Furthermore, enhanced immune activation in the TME was associated with increased chances of survival at 18 months.^99^

This virus proved effective in several preclinical models. Kuryk et al. investigated the cytotoxicity of ONCOS‐102 and pembrolizumab in human melanoma cell lines. The greatest efficiency was demonstrated for ONCOS‐102 plus pembrolizumab, followed by ONCOS‐102 alone, while pembrolizumab did not show any therapeutic benefit by itself. Bodyweight loss and metastasis were not significantly affected by any treatment.^100^ Another study evaluated the antineoplastic activity of combination treatment with standard‐of‐care chemotherapy (pemetrexed, cisplatin, carboplatin) and ONCOS‐102 in a xenograft model of human malignant mesothelioma. The authors demonstrated that ONCOS‐102 was able to induce immunogenic cell death of human mesothelioma cell lines in vitro and showed anti‐tumour activity in the treatment of refractory malignant pleural mesothelioma xenograft model. Additionally, a synergistic anti‐tumour effect was seen when ONCOS‐102 was combined with chemotherapy regimens.^101^

#### 2.3.7 ADV/HSV‐tk

Ad‐mediated expression of herpes simplex virus thymidine kinase (ADV/HSV‐tk) uses an Ad vector to introduce the suicide agent HSV‐tk into tumour cells.^102^ When combined with the prodrug ganciclovir, at the action site, HSV1‐tk phosphorylates it to the monophosphate form, which is further phosphorylated by cellular kinases to the active form, ganciclovir triphosphate, cytotoxic towards proliferating cells.^103^ Suicide gene therapy using the HSV‐tk/ganciclovir system is a well‐studied approach used in cancer therapy.^104^, ^105^, ^106^, ^107^, ^108^ However, there is controversy regarding the combination of HSV‐tk/ganciclovir and replicating Ads, as there are contradictory results if ganciclovir truly improves the oncolytic potential of replicating viruses.^109^, ^110^, ^111^, ^112^, ^113^, ^114^, ^115^, ^116^ ADV/HSV‐tk was generally used in combination with ganciclovir in preclinical studies, a combination that proved effective against hepatocellular carcinoma, breast cancer, melanoma, pituitary tumour and NSCLC models.^103^, ^109^, ^117^, ^118^, ^119^ This combination therapy can be administered safely to cancer patients, the most common adverse event being transient fever and local injection site reaction.^120^

#### 2.3.8 ColoAd1

An interesting proposal for anti‐cancer virotherapy against colon cancer, as an alternative to ONYX‐015 and H101, was ColoAd1 (enadenotucirev), the first oncolytic Ad that was not based on Ad5.^121^, ^122^ Additionally, it has deletions in the E3 and E4 genes, and a chimeric Ad3/Ad11p modification in the E2B gene.^122^ Additionally, due to ColoAd1 having the main capsid proteins similar to Ad11p (which normally bind to CD46), the selectivity of this virus towards colon cancer cells may be because of the overexpressed CD46 receptors on their cellular membrane.^122^, ^123^, ^124^

#### 2.3.9 VCN‐01

VCN‐01 is a novel oncolytic Ad that combines selective replication in Rb‐dysregulated cells, replacement of the heparan sulfate glycosaminoglycan putative‐binding site KKTK of the fibre protein with an integrin‐binding motif RGDK for tumour targeting, and expression of hyaluronidase to degrade the extracellular matrix.^125^ VCN‐01 was tested against a wide range of preclinical cancer models, including pancreatic cancer, melanoma, primitive neuroectodermal tumour, osteosarcoma and glioma.^125^, ^126^, ^127^, ^128^, ^129^

#### 2.3.10 OBP‐301

OBP‐301 is an Ad5‐based oncolytic Ad, which has an hTERT promoter controlling the expression of E1A and E1B genes, driving viral replication. It was tested as monotherapy in liver cancer. The virus showed better local response following intratumoural injection compared to ORR. The better local response was proved by histological assays showing the existence of a necrotic area at the injection site, along with an increased number of CD8^+^ T cells and low levels of PD‐L1. Furthermore, this treatment was proven to have low systemic toxicity, with tolerable adverse events, such as influenza‐like symptoms, fever, fatigue, low platelet count, abdominal distension and anaemia.^130^

#### 2.3.11 CG0070

CG0070 is a conditionally replicating oncolytic Ad whose tumour selectivity is because of the E2F‐1 promoter controlling the expression of the E1A gene. This Ad was designed to express GM‐CSF, to induce anti‐tumoural immunity. This virus was tested on preclinical models of Rb‐defective bladder transitional cell carcinoma. Consequently, it was proved to be more cytotoxic towards cancer cells (by 100 times) compared to normal cells following intratumoural and intravesical administration.^131^ The most frequent mild‐to‐moderate treatment‐related adverse events were proved to be catheter leakage, bladder spasms and dysuria. However, no dose‐limiting toxicities were noticed.^132^

#### 2.3.12 MEM‐288

MEM‐288 is a novel oncolytic Ad with deletions in E1A, E1B and E3 regions of the genome that allows conditional tumour replication.^133^ MEM‐288 carries two potent immune agonists: IFNβ and a recombinant membrane‐stable chimeric form of CD40L (MEM40).^85^ This agonist combination has been demonstrated to be better than either agonist individually to activate conventional dendritic cells type 1, crucial for the enhanced activity of tumour‐antigen reactive CD8^+^ T‐cells.^134^, ^135^, ^136^ Preclinical studies revealed the efficiency of MEM‐288 against ovarian cancer, melanoma and lung cancer models.^137^, ^138^

#### 2.3.13 NG350‐A

NG350‐A is based on enadenotucirev and expresses a full‐length agonistic anti‐CD40 monoclonal antibody, with potential immunomodulating and antineoplastic activities. Upon intratumoural administration of NG‐350A, it selectively targets tumour cells and expresses the agonistic anti‐CD40 antibody, which binds to CD40 on a variety of immune cells from the TME. This activates a cytotoxic T‐lymphocyte‐mediated anti‐tumour immune response and leads to tumour cell death.^139^ The effects of this virus were studied in preclinical models of lung cancer, colorectal cancer and pancreatic cancer, and it was proven to be efficient.^140^

#### 2.3.14 AdAPT‐001

AdAPT‐001 is an attenuated human Ad vector, TAV‐255, modified to express a human TGF‐β trap fusion protein that neutralises TGF‐β, with potential oncolytic, immunomodulating and antineoplastic activities. Upon administration, AdAPT‐001 selectively induces tumour cell lysis, releasing a variety of tumour‐associated antigens, which may eventually activate a systemic immune response against the cancer cells.^141^

#### 2.3.15 CAdVEC

**FIGURE 3:**
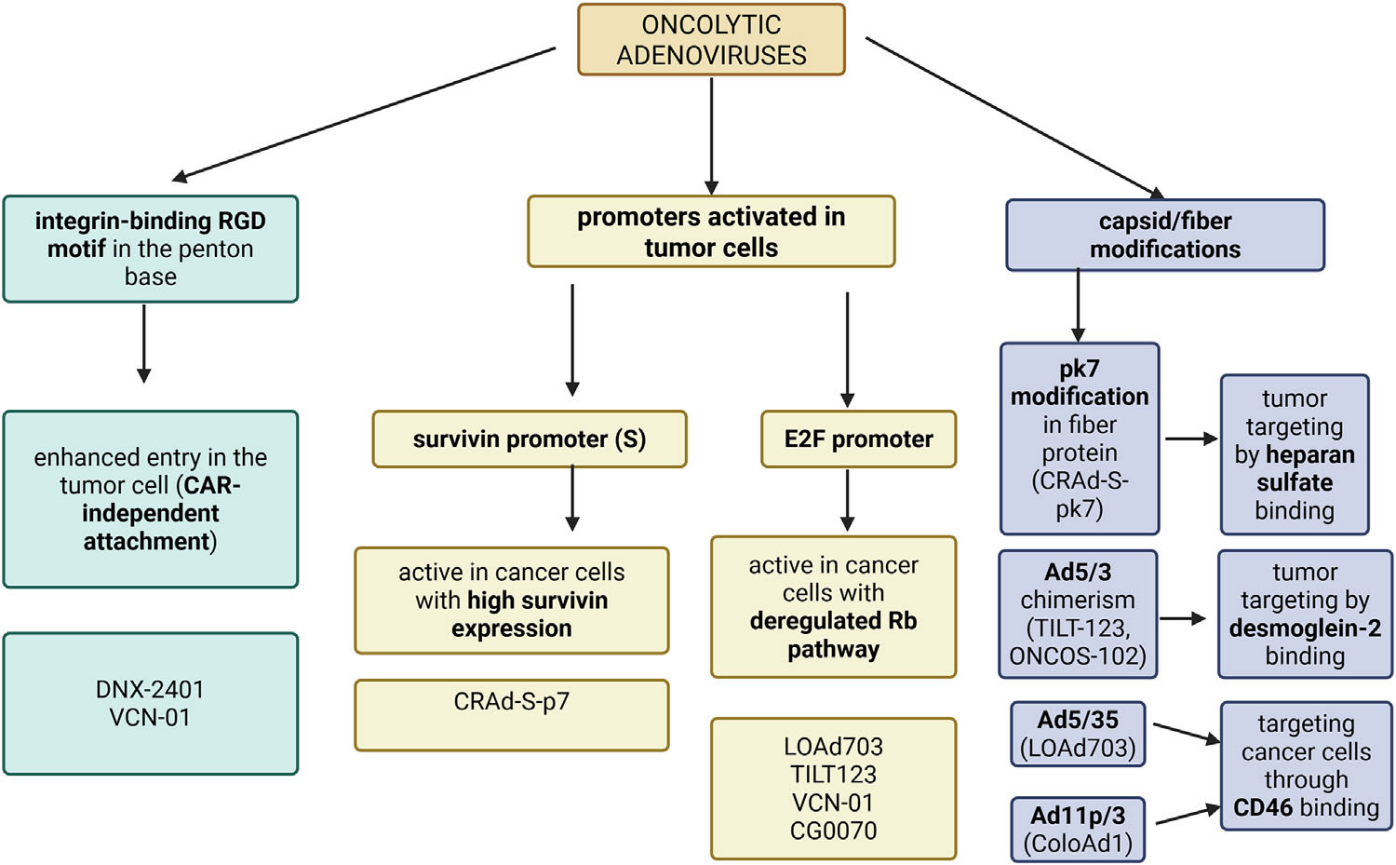
Tumour‐targeting strategies in oncolytic adenoviruses. Entry‐level modifications include RGD insertion in the penton base to enable integrin‐mediated infection and chimeric fibre/capsid alterations (e.g., polylysine insertion or fibre swapping) that redirect viral attachment away from native CAR dependence toward tumour‐associated receptors such as heparan sulfate proteoglycans, desmoglein‐2 and CD46. Transcriptional targeting strategies restrict viral replication to cancer cells using tumour‐selective promoters, including the survivin promoter, active in survivin‐overexpressing malignancies such as glioma and ovarian cancer, and the E2F promoter, which drives replication in cells with deregulated Rb/E2F signalling. Together, these approaches enhance tumour selectivity by combining improved cellular entry with cancer‐restricted viral gene expression and replication. Created in BioRender, Iova V. (2026); https://BioRender.com/f1ru3p0.

CAdVEC is a binary oncolytic/helper‐dependent Ad system combining an oncolytic Ad with a helper‐dependent Ad (HDAd). The HDAd is nonlytic and has no viral genes, with a cargo capacity of up to 34 kb, allowing the virus to carry multiple immunomodulatory molecules, enabling more durable anti‐tumour activity.^64^, ^142^ This platform was tested on preclinical cancer models such as breast cancer and NSCLC.^64^, ^143^ Figure 3 presents various tumour targeting strategies across different Ads.

### 2.4 Oncolytic Ads in combination therapies—Clinical trials

Multiple clinical trials employing oncolytic Ads combined with chemotherapy, radiotherapy, immunotherapy or targeted therapy were conducted over the years (Table 1A, 1B).

**TABLE 1A:** Completed trials with reported results.

Ad	Viral backbone	Deleted genes	Added genes	Types of cancer	References	Combination	Main findings
H101	Ad5	E1B‐55K parts of the E3 region	Does not carry an added transgene	Refractory/recurrent gynaecological malignancies	NCT05051696 (Phase 2)^144^	Radiotherapy	Interim outcomes 116 enrolled patients: 100 evaluable ORR: 64% DCR: 79% Median PFS: 8.7 months Adverse events: 83% (fever, fatigue and injection‐site pain)^144^
LOAd703	Ad5/35	E1A CR2 E3‐6.7K E3 gp19K	E2F promoter for E1A TMZ‐CD40L 4‐1BBL Chimeric fibre modification (Ad5/35)	Pancreatic cancer	NCT02705196 (Phase 1/2)^145^	Chemotherapy (gemcitabine + nabpaclitaxel) atezolizumab (PDL1 inhibitor)	Results published for Arm 1 (LOAd703 plus chemotherapy) 18 evaluable patients ORR: 44% Increase in the CD8^+^ effector memory cells and Ad‐specific T cells—in 15 (94%) of 16 evaluable patients^145^
CRAd‐S‐p7	Ad5	Natural E1A promoter	Survivin promoter for E1A Polylysine (pK7) modification to fibre protein	Malignant glioma	NCT03072134 (Phase 1)^146^	Radiotherapy Chemotherapy (temozolomide)	12 patients Partial response: 8% Pseudoprogression: 8% Stable disease: 84% Median PFS: 9.05 months Median OS: 18.4 months^146^
DNX‐2401	Ad5	E1A CR2	RGD motif in fibre protein	Recurrent glioblastoma or gliosarcoma	NCT02197169 (Phase 1)^87^	IFN‐γ	27 patients OS‐12: 33% OS‐18: 22%^87^
					NCT02798406 (Phase 2)^147^	Pembrolizumab (PD‐1‐blocking antibody)	49 patients ORR: 10.4% OS‐12: 52.7% Median OS: 12.5 months DCR: 56.2%^147^
					NCT01956734 (Phase 1)^148^	Chemotherapy (temozolomide)	31 glioma patients at first recurrence No severe toxicities Three patients alive 30, 19 and 27 months after treatment^149^
TILT‐123	Ad5/3	24 bp in the E1A CR2 region	TNF α IL‐2 E2F promoter for E1A Ad5/3 fibre knob chimerism	Metastatic melanoma	NCT04217473 (Phase 1)^150^, ^151^	TILs	17 evaluable patients ORR: 11.7% DCR: 35% Median OS: 447 days^150^, ^151^
				Ovarian cancer	NCT05271318 (Phase 1a/1b)^152^	Pembrolizumab (Phase 1a) Pembrolizumab and chemotherapy (pegylated liposomal doxorubicin) (Phase 1b)	Results only for Phase 1a 14 evaluable patients DCR: 64% Median PFS: 98 days Median OS: 190 days^152^
ONCOS‐102	Ad5/3	24 bp in the E1A CR2 region	Ad5/3 fibre chimeric protein GMCSF	Advanced solid tumours	NCT01598129 (Phase 1)^98^	Chemotherapy (cyclophosphamide)	12 enrolled patients 10 evaluable patients DCR: 40% Median OS: 9.3 months^98^
				Pleural mesothelioma	(Phase 2)^99^	Chemotherapy (pemetrexed and cisplatin/carboplatin)	31 enrolled patients Anaemia and neutropenia were the most frequent severe adverse events No statistically significant difference in efficacy for the combination therapy vs. chemotherapy alone 30‐month OS: 34.1% for the combination therapy vs. 0% for chemotherapy alone Median OS: 20.3 months for combination therapy vs. 13.5 months for chemotherapy alone^99^
ADV/HSV‐tk	Ad5	Not specified	HSVtk	TNBC and metastatic NSCLC	NCT03004183 (Phase 2)^102^	SBRT Valacyclovir Pembrolizumab	Results for NSCLC patients 28 enrolled patients 27 evaluable patients ORR: 33.3% CBR: 70.4% Clinical benefits for ICPIs‐refractory patients: 75% Median PFS: 7.4 months Median OS: 18.1 months^102^
ColoAd1	Ad11p/3	In the E3 region and parts of the E4 region	Ad11p/3 chimerism	Locally advanced rectal cancer	NCT03916510 (Phase 1)^153^	Chemoradiotherapy	13 enrolled patients 12 evaluable patients Acceptable safety profile with evidence of a higher‐than‐expected rate of response by mrTRG^153^
VCN‐01	Ad5	24 bp in the E1A CR2 region Partial E3 deletion	Human PH20 hyaluronidase E2F‐1 promoter for E1A RGD motif in the fibre protein	Advanced solid tumours (pancreatic adenocarcinoma)	NCT02045602 (Phase 1)^154^	Chemotherapy (nabpaclitaxel/gemcitabine)	42 enrolled patients, of whom 26 used combination therapy For pancreatic adenocarcinoma ORR: 50%^154^
				Advanced pancreatic cancer	NCT02045589 (Phase 1)^126^		8 patients Disease stabilisation Virus replication Stromal disruption^126^
					NCT05673811 (Phase 2)^155^		96 enrolled patients Median OS: 10.8 months in combination regimen group vs. 8.6 months in chemotherapy group Median PFS: 7.0 months in combination regimen group vs. 4.6 months in chemotherapy group ORR: 19/48 patients in combination regimen group vs. 15/48 in chemotherapy group DCR‐37/48 48 patients in combination regimen group vs. 34/48 in chemotherapy group^155^
MEM‐288	Ad5	In E1A, E1B and E3 regions	IFNβ CD40L	Advanced solid tumours (NSCLC)	NCT05076760 (Phase 1)^156^	Nivolumab (anti‐PD1 monoclonal antibody) Chemotherapy (docetaxel)	Results for monotherapy on 14 NSCLC patients Shrinking in 45% of evaluable patients^156^
NG350‐A	Ad11p/Ad3	Not specified	Agonist anti‐CD40 monoclonal antibody	Advanced or metastatic epithelial tumours	NCT03852511 (Phase 1)^157^	ICPIs	Results for monotherapy on 16 No cases of Grade ≥3 adverse events Increased serum IL‐12 and IFNγ T‐cell clones expansion^157^
AdAPT‐001	Ad5	50 bp in the E1A promoter region E1B‐19K	Chimeric transforming growth factor β (TGFβ) receptor II–Fc fusion protein	Refractory solid tumours	NCT04673942 (Phase 2)^158^	ICPIs	Results of monotherapy on 12 advanced soft tissue sarcoma patients Durable stable disease Shrinkage of both injected and non‐injected lesions Downstaging Response to ICPIs^158^
CAdVEC	Not specified	All viral genes deleted for HDAd 24‐bp deletion from E1A for OAd	IL‐12p70 (HDAd) Anti–PD‐L1 antibody (HDAd) HSVtk (HDAd)	Advanced HER2‐positive solid tumours	NCT03740256 (Phase 1)^64^	HER2‐specific autologous CAR T cells	Results for 4 patients treated only with CAdVEC, followed by pembrolizumab Increased levels of Th1‐ and Th2‐associated cytokines Transient systemic increases in cytokines No circulating viruses in blood, urine or buccal swab samples 3 of 4 tumour responses^64^
CG0070	Ad5	Natural E1A promoter	E2F‐1 promoter for E1A GM‐CSF	Bladder cancer	NCT04610671 (Phase 1)^132^	ICPI (nivolumab)	21 patients enrolled and treated No dose‐limiting toxicity Pathological complete response rate: 42.1% 1‐year recurrence‐free survival rate: 70.4%

Abbreviations: CAR T cells, chimeric antigen receptor T cells; DCR, disease control rate; HAIC, hepatic artery infusion chemotherapy; HER2, human epidermal growth factor receptor 2; ICPI, immune checkpoint inhibitor; NSCLC, non‐small cell lung cancer; ORR, objective response rate; OS, overall survival; PD‐1, programmed death‐1; PD‐L1, programmed death‐ligand 1; PFS, progression‐free survival; SBRT, stereotactic body radiotherapy; TILs, tumour‐infiltrating lymphocytes; TNBC, triple‐negative breast cancer.

**TABLE 1B:** Clinical trials with no results reported and their status.

Ad	Types of cancer	References	Combination	Expected completion date/status
H101	Advanced pancreatic cancer	NCT06196671 (Phase 2)^159^	Camrelizumab (PD‐1‐blocking antibody)	1 January 2028 (ongoing)
	Advanced malignant pleural mesothelioma	NCT06031636 (Observational)^160^	PD‐1 inhibitor	31 July 2026 (ongoing)
	Advanced hepatocellular carcinoma	NCT05113290 (Phase 4)^161^	Sorafenib (targeted therapy)	1 December 2023 (unknown status)
	Recurrent cervical cancer	NCT05234905 (Phase 2)^162^	Camrelizumab	December 2024 (unknown status)
	Advanced biliary tract cancer	NCT06919848 (Phase 2)^163^	Lenvatinib (targeted therapy) Toripalimab (PD‐1‐blocking antibody)	1 May 2027 (ongoing)
		NCT05124002 (Phase 4)^164^	HAIC	1 April 2026 (unknown status)
	Colorectal adenocarcinoma liver metastases	NCT07381309 (Phase 2)^165^	SBRT PD‐1 monoclonal antibody chemotherapy targeted therapy	1 February 2029 (ongoing)
CRAd‐S‐p7	Malignant glioma	NCT05139056 (Phase 1)^166^	Surgical resection	Suspended
TILT‐123	Metastatic melanoma	NCT06961786 (Phase 1)^167^	Lymphocyte‐depleting chemotherapy TILs	April 2027 (ongoing)
	Advanced solid tumours (melanoma or head and neck squamous cell carcinoma)	NCT05222932 (Phase 1)^168^	Avelumab (anti‐PD‐L1 antibody)	December 2026 (ongoing)
ONCOS‐102	Advanced treatment‐resistant melanoma	NCT05561491 (Phase 2)^169^	Balstilimab (a PD‐1 inhibitor)	June 2027 (withdrawn)

Abbreviations: CAR T cells, chimeric antigen receptor T cells; DCR, disease control rate; HAIC, hepatic artery infusion chemotherapy; HER2, human epidermal growth factor receptor 2; ICPI, immune checkpoint inhibitor; ORR, objective response rate; OS, overall survival; PD‐1, programmed death‐1; PD‐L1, programmed death‐ligand 1; PFS, progression‐free survival; SBRT, stereotactic body radiotherapy; TILs, tumour‐infiltrating lymphocytes.

#### 2.4.1 Combination with immunotherapy

By far, oncolytic Ads were tested in combination with immunotherapy (ICPIs, TILs, CAR‐T cells or IFN approaches) against various malignancies.

H101 was most frequently combined with ICPIs (such as camrelizumab and toripalimab) (NCT06196671, NCT06031636, NCT05234905, NCT06919848, NCT07381309).^159^, ^160^, ^162^, ^163^, ^165^ This shows that H101 is studied as a promising approach to reverse cancer resistance to ICPIs. However, these studies are still ongoing, and no results have been reported.

The OS‐12 for the combination of DNX‐2401 with IFN‐γ (or for DNX‐2401 alone) was 33%, while for the combination of DNX‐2401 with pembrolizumab, 52.7%.^87^, ^147^ It has been previously reported that the OS‐12 rate was 36% (95% confidence interval [CI]: 32%–40%) for re‐irradiation against recurrent glioblastoma.^88^ These results suggest that DNX‐2401 followed by pembrolizumab can bring notable survival benefits, but further studies are required to study the exact mechanism of action. The difference in OS‐12 between combinations likely reflects how each partner therapy interacts with DNX‐2401–induced anti‐tumour immunity. DNX‐2401 already promotes tumour cell lysis and can stimulate immune activation.^85^ When combined with pembrolizumab, a PD‐1 inhibitor, this may more effectively sustain and amplify T‐cell responses by preventing exhaustion, leading to stronger and more durable systemic immune control and thus higher OS‐12.^170^ In contrast, IFN‐γ may enhance local immune activation, but can also have pleiotropic and context‐dependent effects, including potential immunoregulatory feedback or limited ability to overcome tumour‐induced T‐cell exhaustion, resulting in less consistent survival benefit.^171^, ^172^ Overall, variability in OS‐12 across combinations likely reflects differences in the ability to convert virus‐induced immunogenicity into a sustained systemic anti‐tumour immune response, rather than differences in viral oncolysis itself. Additionally, in terms of adverse effects, the combination was well‐tolerated and caused no dose‐limiting toxicities.^147^ Nevertheless, even though the OS‐12 for DNX‐2401 with IFN‐γ (or for DNX‐2401 alone) was lower than the determined value for re‐irradiation therapy, the mechanism of action of the combination is worth studying, as out of three patients who remained alive at 19, 21 and 22 months, two received the DNX‐2401 with IFN‐γ, and only one received DNX‐2401 alone.^87^

Regarding TILT‐123, the most common combination partner investigated for this virus was immunotherapy, either TILs (NCT06961786, NCT04217473) or ICPIs (NCT05222932, NCT05271318).^150^, ^151^, ^152^, ^167^, ^168^ In terms of adverse events, the combination approaches were well tolerated, and no dose‐limiting toxicities were observed, the most frequent mild‐to‐moderate adverse events being fever, fatigue and nausea.^150^, ^152^ TILs represent a novel form of immunotherapy approved by the FDA for metastatic melanoma. TILT‐123 was combined with TILs‐based therapy to replace the toxic pre‐ and post‐conditioning regimens (lymphodepleting chemotherapy and high‐dose IL‐2 regimens, respectively).^150^, ^151^ At first, the results from NCT04217473 appear not more promising (ORR = 11.7%, DCR = 35%) compared to other studies, which tested different approaches against ICPIs‐resistant melanoma. For instance, Acar et al. showed that treatment with anti‐PD‐1 against anti‐CTLA‐4‐resistant melanoma led to an ORR of 42.9% and a DCR of 53%, while treatment with nivolumab–ipilimumab against anti‐PD‐1‐resistant melanoma resulted in an ORR of 17.9% and a DCR of 25%.^173^ However, when comparing these results, several factors should be taken into consideration. First, only 17 patients were included in NCT04217473, while 56 patients divided into two equal groups were included in the study of Acar et al. Second, the patients from NCT04217473 were heavily pretreated (range of one to seven prior cancer treatment lines), while over 50% of the patients from Acar et al. received the second line of treatment, and no patient received more than four lines of treatment.^150^, ^173^ Thus, further trials based on TILT‐123 and TILs must be conducted using a greater number of patients for more clinical relevance. The combination of this Ad with pembrolizumab showed really promising results on patients with ovarian cancer, as the DCR (which was 64% in NCT05271318) was higher than the DCR noticed in trials investigating single‐agent ICPIs (ranging from 23% to 45%^152^, ^174^, ^175^). Nevertheless, the low number of evaluable patients (14) demands further trials employing this combination for better clinical significance.

Shoushtari et al. investigated intratumoural ONCOS‐102 plus anti–PD‐1 therapy in anti–PD‐1–resistant melanoma in 21 patients, obtaining an ORR of 35%, which is almost double than the ORR obtained for treating this type of melanoma with nivolumab–ipilimumab (17.9%), as described by Acar et al.^173^, ^176^ Thus, ONCOS‐102 might indeed represent an immunosensitising agent for combinatory therapies with ICPIs, as suggested by NCT01598129.^98^ Studies showed that ONCOS‐102 was safe and well tolerated, the majority of adverse events being pyrexia, chills and nausea.^98^, ^176^

As for ADV/HSV‐tk, in NCT03004183, the overall ORR for treating metastatic NSCLC with ADV/HSV‐tk and valacyclovir, SBRT and pembrolizumab was 33.3%.^102^ However, in order to compare it with pembrolizumab monotherapy, it is important to determine the specific ORR depending on the tumour PD‐L1 expression, and Guan et al. determined the following ORRs: 50% for PD‐L1 over 50%, 40% for PD‐L1 1%–49% and 26.7% for PD‐L1 0%.^102^ For ICPIs monotherapy, the ORR ranges from 35% to 43% in patients with PD‐L1 over 50%, from 13% to 20% in patients with PD‐L1 1%–49% and is under 10% in patients with PD‐L1 under 1%.^177^ As it can be noticed, ORRs across all groups were higher for the combination therapy compared to ICPIs monotherapy, but the highest benefit was noticed for tumours expressing between 1% and 49% PD‐L1, while the lowest gain was for tumours expressing over 50% PD‐L1. This is in accordance with prior studies, which stated that the largest benefit from the addition of radiotherapy to pembrolizumab was in patients with PD‐L1‐negative NSCLC.^178^ This may be due to the fact that radiotherapy can induce upregulation of PD‐L1 expression, enhancing the therapeutic effect of anti‐PD‐1/PD‐L1 inhibitors.^121^ Thus, the overall effect of PD‐L1 upregulation might have less influence on the sensitivity of tumours expressing already high levels of PD‐L1 compared to tumours expressing lower levels of this molecule. Further studies are required to explain this behaviour.

MEM‐288 is another oncolytic Ad with the potential of being a partner for chemotherapy or ICPIs in the treatment of NSCLC refractory to ICPIs and platinum compounds. Saltos et al. showed that intratumoural injection of MEM‐288 was safe, with mild‐to‐moderate injection site reactions and flu‐like symptoms, and also due to tumour shrinkage associated with the generation of an immune‐active tumour microenvironment and the activation of systemic immunity.^156^

Moreover, NG‐350A was proven to be effective against advanced epithelial tumours after intravenous administration. Most importantly, this effectiveness is associated with less severe adverse events compared to the other intravenously administered viruses we discussed previously. Thus, the increased and prolonged anti‐tumoural immune response, stability in circulation and reduced toxicity make NG‐350A a very promising candidate for further clinical studies, and combining it with other immunotherapies, such as ICPIs, ought to be taken into consideration for an even more enhanced anti‐tumoural effect.^157^

AdAPT‐001 was found to have promising effects on various soft tissue sarcomas. It is worth mentioning that one patient with leiomyosarcoma presented a tumour shrinkage of 72.2%, which is higher than other therapies.^158^ For instance, one case report presented a size decrease of a primary pulmonary leiomyosarcoma from 11 to 5.6 cm (so a 49.1% decrease in tumour size) following chemotherapy using doxorubicin.^179^ Another case report presents a 31% decrease in tumour size at 1 month and a 21% at 3 months after high‐intensity focused ultrasound ablation of a presumed uterine leiomyomata that was later diagnosed as a uterine leiomyosarcoma.^180^ So, AdAPT‐001 might have a more potent anti‐tumoural effect compared to other therapies, but future trials are required as the data we presented are from case reports.

Regarding CAdVEC, Wang et al. developed an interesting combination therapy of ultralow‐dose binary oncolytic/helper‐dependent Ad (ULCA) and ICPIs (pembrolizumab) in patients with advanced solid tumours refractory to standard treatments. The aspect worth highlighting is that the used dosage of oncolytic virus was more than 100‐fold lower than in other clinical trials, leading to few and low‐grade adverse events. Additionally, the low dose leads to a lack of oncolytic Ads in blood, urine or buccal swab samples. Three of four patients treated with a single ULCA intratumoural injection showed tumour responses, including one surgically confirmed complete response, and abscopal effects in untreated metastatic tumours. Even at extremely low doses, this platform was sufficiently potent to induce significant tumour control through oncolysis and immune repolarisation by delivering both an oncolytic Ad and a helper‐dependent Ad expressing IL‐12p70, anti–PD‐L1 antibody, and HSVtk safety switch, enhancing tumour‐infiltrating T‐cell activity and proliferation.^64^ Thus, CAdVEC represents an alternative for patients with injectable tumour lesions, especially for those not tolerating the more severe adverse effects of other therapies, including regimens involving systemic oncolytic viruses. However, due to the low number of patients in this trial, future studies are required, including those involving combination with different therapies.

#### 2.4.2 Combination with conventional therapies—Chemotherapy, radiotherapy and surgical resection

A number of clinical trials have studied the potential of combining oncolytic Ads with conventional anti‐cancer therapies, such as chemotherapy, radiotherapy or surgical resection.

H101 was studied along with chemotherapy (NCT05124002) and radiotherapy (NCT05051696).^144^, ^164^ Interim results for NCT05051696 showed that the combination of H101 intratumoural administration and radiotherapy in refractory or recurrent gynaecological malignancies showed promising results, suggesting that the therapy was efficacious and safe.^144^ The effectiveness of H101 was associated with increased B cells and M1 tumour‐associated macrophages in the tumour microenvironment.^144^

Regarding LOAd703, in the clinical trial NCT02705196, results were published only for Arm 1 of the trial (administration of LOAd703 plus chemotherapy). The ORR was 44%, which is higher than the ORR in patients with advanced pancreatic ductal adenocarcinoma after first‐line treatment with gemcitabine and nabpaclitaxel (34.4%).^145^, ^146^ Additionally, it is worth noticing that the ORR obtained in this trial is greater than the ORR for FOLFIRINOX (oxaliplatin, irinotecan, fluorouracil and leucovorin) chemotherapy of pancreatic cancer, which was proven to determine the highest ORR for pancreatic cancer (38.3%).^74^ The benefit of LOAd703 addition may be due to increased infiltration with T cells in the tumour microenvironment, but further research must study its mechanism of action. Additionally, the treatment was safe; the most common mild‐to‐moderate adverse events attributed to LOAd703 were fever, chills and elevated liver enzymes.^145^

Studies investigated the combination of CRAd‐S‐pk7 delivered via an NSC line with radio‐ and chemotherapy (NCT03072134) and with surgical resection (NCT05139056).^146^, ^166^ We found results only for NCT03072134.^146^ The Stupp protocol, the standard of care against newly diagnosed glioblastoma, involving radiation therapy and temozolomide, leads to a median OS of 14.6 months.^181^ According to the results from NCT03072134, the combination of CRAd‐S‐pk7 delivered via an NSCLC line and chemoradiotherapy led to a median OS of 18.4 months, suggesting its efficiency compared to standard therapy.^146^ Moreover, the treatment was safe and non‐toxic. The adverse events were not related to virotherapy, and all were commonly observed toxicities due to subsequent chemo‐ and radiotherapy.^146^

DNX‐2401 has been studied in combination with chemotherapy (temozolomide) (NCT01956734) in the treatment of brain tumours. From the results reported so far, three out of 31 glioma patients at first recurrence were alive at 19, 27 and 30 months, respectively, following the combination regimen, with no severe toxicities reported.^149^ It has been previously reported that the median OS for patients with recurrent or progressive glioblastoma following radiotherapy and temozolomide was 6 months.^182^ Thus, even though the median OS for NCT01956734 has not been reported, given the fact that three patients were able to reach more than 19 months of survival following the combination regimen, DNX‐2401 plus temozolomide has the potential to lead to a more efficient anti‐tumoural response. Further studies are required to study this combination approach.

As for ColoAd1, the trial NCT03916510 demonstrated that systemic administration of this virus, together with chemoradiotherapy in advanced local cancer, resulted in mainly mild‐to‐moderate adverse events, the most common being gastrointestinal ones. However, at increased dosing regimens, two observed dose‐limiting toxicities were noticed, mainly leg swelling and acute kidney injury.^153^ Even though this study presented a higher‐than‐expected rate of response and suggested a possible radiosensitising potential for ColoAd1, the systemic toxicities associated with intravenous administration of the virus at higher doses are not to be put aside, and further studies are required to find delivering platforms or the specific doses at which the efficiency is the highest with tolerable toxicities.

Regarding VCN‐01, clinical trials are focusing mainly on the combination with chemotherapy (nabpaclitaxel/gemcitabine) against pancreatic adenocarcinoma (NCT02045602, NCT02045589, NCT05673811).^126^, ^154^, ^155^ We wanted to compare the efficiency of intravenous (NCT02045602) versus intratumoural (NCT02045589) administration of VCN‐01 in this combination therapy. The apparent higher efficacy of intravenous VCN‐01 compared with intratumoural administration is mainly due to its systemic biodistribution, allowing the virus to reach both injected tumours and distant, non‐injected metastatic lesions, whereas intratumoural delivery is spatially limited to accessible lesions and may not control occult disease. In contrast, the greater toxicity observed with intravenous administration reflects its widespread systemic exposure, leading to increased interaction with normal tissues and immune‐related adverse events, while intratumoural injection results in more localised exposure and consequently a better safety profile. Thus, the differences between the two routes reflect a trade‐off between systemic anti‐tumour coverage and treatment‐related toxicity, rather than differences in intrinsic viral potency (Table 2).^126^, ^154^

**TABLE 2:** Comparison of intravenous versus intratumoural VCN‐01 administration in clinical efficacy, durability and safety.

Parameter	Intravenous administration (NCT02045602)	Intratumoural administration (NCT02045589)
ORR	50% (5/10 or 6/12 evaluable patients, depending on schedule)	Not explicitly reported; tumours are mostly stable or reduced
Disease control duration	Long stabilisations >1 year in responders; one >4 years survival	Mostly progression by 4 months; rare longer control (up to 31 months in 1 patient)
Disease progression pattern	Better systemic control, fewer distant failures implied	Frequent progression at 4–8 months; likely due to untreated metastatic sites
Safety profile	Higher toxicity (Grade 4–5 events: AST increase, febrile neutropenia, thrombocytopenia, enterocolitis)	Lower toxicity (no adverse events >Grade 3)
**Clinical implication**	**Preferred for metastatic/systemic disease**	**Preferred for localised tumours**

Abbreviations: AST, aspartate aminotransferase; ORR, objective response rate.

#### 2.4.3 Combination with targeted therapy

Although combinations of oncolytic Ads with targeted therapies are conceptually promising, clinical validation remains limited. Trials such as H101 in combination with sorafenib for advanced hepatocellular carcinoma (NCT05113290) and with lenvatinib in advanced biliary tract cancer (NCT06919848) are currently ongoing, reflecting growing interest in integrating OVs with anti‐angiogenic and kinase‐inhibiting agents.^161^, ^163^ The rationale for these approaches lies in the potential for targeted therapies to modulate the TME—particularly through vascular normalisation and suppression of pro‐tumoural signalling pathways—thereby enhancing viral delivery, replication and immune activation.^183^, ^184^ Conversely, OVs may potentiate the efficacy of targeted agents by promoting immunogenic cell death and increasing tumour immunogenicity.^12^ However, the absence of published clinical outcomes from these studies highlights a persistent gap between preclinical synergy and clinical translation. Key questions remain regarding optimal dosing schedules, sequencing strategies and the management of overlapping toxicities. As these trials mature, their results will be critical for determining whether such combinations can achieve meaningful improvements in patient outcomes or whether additional optimisation of OV platforms and patient selection will be required.

## 3 CORE CHALLENGES OF ONCOLYTIC ADS IN COMBINATION CANCER THERAPY

A central challenge for OVs in combination cancer therapy remains the balance between systemic efficacy, safety and efficient tumour targeting. As illustrated by prior discussion of VCN‐01, intravenous delivery can improve anti‐tumoural activity in metastatic settings, but is frequently accompanied by increased systemic toxicity, whereas intratumoural administration offers a safer profile at the cost of limited control over distant lesions.^126^, ^154^ This trade‐off reflects a broader limitation in the field: many approved OVs still rely on intratumoural injection, restricting their applicability in disseminated disease. Even with next‐generation platforms such as TILT‐123, which incorporates capsid modifications and immunostimulatory transgenes to enable intravenous use and enhance T‐cell recruitment, achieving consistent and sufficient intratumoural viral accumulation remains difficult, as increased dosing does not necessarily translate into higher tumour uptake.^95^ Moreover, the immunosuppressive tumour microenvironment and rapid immune clearance further constrain therapeutic efficacy. Emerging strategies, including biomaterial‐based delivery systems like calcium phosphate‐camouflaged, enzyme‐engineered outer membrane vesicles, aim to overcome these barriers by prolonging circulation, enhancing tumour homing and amplifying virus‐induced mechanisms such as autophagy.^44^ However, these approaches also highlight additional complexities, including controlling oxidative stress, ensuring selective activation within tumours, and maintaining safety. Collectively, these issues underscore that improving delivery efficiency, minimising systemic toxicity and effectively reshaping the tumour microenvironment are still key hurdles limiting the full potential of OVs in combination therapy

## 4 CONCLUSIONS AND FUTURE PERSPECTIVES

In conclusion, the integration of oncolytic Ads into modern oncology represents a shift toward multimodal therapeutic strategies. By categorising these agents based on their functional synergy, we can observe a clear trend: they are no longer viewed merely as standalone lytic agents, but as powerful sensitisers and immunomodulators. Viruses like H101, LOAd703 and ColoAd1 act as potent adjuncts to standard radiotherapy and chemotherapy, particularly in recalcitrant cases like pancreatic and gynaecological cancers. Agents such as DNX‐2401, ONCOS‐102 and ADV/HSV‐tk are proving vital in ‘heating up’ cold tumours, allowing ICPIs to be effective even in previously resistant melanomas and glioblastomas. Innovations in delivery—such as the NSC lines used for CRAd‐S‐pk7 or the ultralow dosage dual‐platform of CAdVEC—address the historical challenges of systemic toxicity and blood–brain barrier penetration. TILT‐123 stands out for its potential to revolutionise TIL‐based therapies by eliminating the need for toxic conditioning regimens, thereby improving the patient's quality of life during treatment.

The regimens that employed intratumoural administration of the virus were generally better tolerated compared to intravenous administration.

Nevertheless, the majority of the included trials were of early phase and were conducted on a limited number of patients. Thus, they have to be included in further clinical trials to better understand their safety profiles, long‐term adverse reactions and anti‐tumoural effects on cancers in order to be approved for clinical use.

## AUTHOR CONTRIBUTIONS

Conceptualisation: V.I., G.M.I., M.S.T., I.S. and C.G.S. Methodology: V.I. and I.S. Writing—original draft preparation: V.I., G.M.I., M.S.T., I.S. and C.G.S. Writing—review and editing: V.I., G.M.I., M.S.T., I.S. and C.G.S. Supervision: M.S.T.

## CONFLICT OF INTEREST STATEMENT

The authors declare no conflicts of interest.

## ETHICS STATEMENT

Not applicable.
